# Knowledge of Cancer Genetics and the Importance of Genetic Testing: A Public Health Study

**DOI:** 10.7759/cureus.43016

**Published:** 2023-08-06

**Authors:** Nasser Al Shanbari, Abdulrahman Alharthi, Salah M Bakry, Muath Alzahrani, Majed M Alhijjy, Hashem A Mirza, Meshal Almutairi, Samar N Ekram

**Affiliations:** 1 Department of Medicine and Surgery, College of Medicine, Umm Al-Qura University, Mecca, SAU; 2 Department of Clinical Nutrition, Maternity and Children Hospital, Buraidah, SAU; 3 Department of Medical Genetics, College of Medicine, Umm Al-Qura University, Mecca, SAU

**Keywords:** inheritance, public health, genetic testing, cancer, awareness

## Abstract

Background

Cancer represents a global concern as the second-leading cause of mortality worldwide. It is defined as a genetic disease that develops as a result of several genetic abnormalities and changes to specific genes. Thus, early preventive measures and clinical interventions can be implemented with impressive results using genetic testing and screening for hereditary susceptibility.

Objectives

The present study assessed the knowledge of cancer genetics and of the importance of genetic testing among the general population in Saudi Arabia’s Makkah Province.

Methods

A descriptive cross-sectional study was conducted among the general population in Makkah Province. The data were collected through an online questionnaire from November 2022 through December 2022.

Results

The study recruited 1,329 participants, the largest group of whom were 21-30 years old (n=524, 39.4%). About 60.1% of the respondents were female. The findings reveal that 52.52% of the respondents had poor knowledge, while only 4.82% exhibited good knowledge.

Conclusion

Approximately half the total participants possessed an inadequate understanding of cancer genetics and the importance of genetic testing. This indicates the need for awareness campaigns and programs to improve the general population’s understanding of the genetic predisposition to cancer.

## Introduction

Cancer represents a global concern, as it is responsible for more than 9.6 million deaths annually and is the second-leading cause of death worldwide [1]. In 2018, the Kingdom of Saudi Arabia (KSA) recorded 10,518 cancer deaths and 24,485 new cases [2]. The most prevalent types of cancer reported in KSA are breast, colorectal, prostate, brain, lymphoma, kidney, and thyroid [3].

Cancer is considered a genetic disease and results from a number of genetic changes and mutations in certain genes [4,5]. Cancer genes are classified into three types [4,5]. Oncogenes induce uncontrolled cell growth through gain of function, while the second type, tumor suppressor genes, lose their normal function of suppressing cell growth. The third type, DNA repair genes, allow errors to accumulate when they are defective, which can lead to cancer formation [4,5].

The prevalence of various types of cancer has increased threefold over the past 10 years as reported in a review study conducted in the KSA [3]. This increase may be influenced by multiple factors, such as a lack of cancer awareness, a lack of screening and early detection programs, social barriers related to cancer investigations, and genetic predisposition to cancer [3].

Hereditary cancer syndrome occurs when a person inherits certain gene(s) mutation(s), increasing the risk of developing certain tumors at a relatively young age [6]. There are numerous types of hereditary cancer syndromes, including hereditary breast and ovarian cancer syndrome, Lynch syndrome (formerly called hereditary nonpolyposis colorectal cancer), and others [7]. A recent study reports that inherited genetic defects cause of 10% of all cancers [7].

Genetic testing and screening for hereditary susceptibility can promote the implementation of early preventive measures and clinical interventions with significant outcomes [8]. It is essential that patients adequately understand the importance of genetic testing, as it may facilitate the acceptance of tailored medical treatments and support early detection and prevention [9]. Conversely, a low level of societal awareness of the significance of genetic testing could limit cancer prevention and lead to unnecessary treatment and surgical interventions [9]. For that reason, we assessed the knowledge of cancer genetics and of the importance of genetic testing among a general population.

## Materials and methods

Study design and participants

A descriptive cross-sectional survey-based study was conducted from November 16 through December 16, 2022. Individuals aged 18-65 years who lived in Mecca Province were invited to participate in the study. We excluded those who were physically ill or incapable of communicating with field researchers and those who declined to participate.

Sample size and ethical considerations

According to the General Authority for Statistics in Saudi Arabia, Mecca’s estimated population in 2017 was 1,908,000. We calculated the sample size using OpenEpi software [10]. To obtain 95% confidence and a 5% acceptable error margin for the Mecca province population [11], a study design effect of two and a 10% added sample to account for incomplete participation required 486 participants.

The Umm Al-Qura Ethical Committee granted ethical approval for this survey in November 2022 (approval number HAPO-02-K012-2022-11-1269), and the study adhered to the principles of the Declaration of Helsinki. We distributed our questionnaire to 1,329 participants through convenience sampling.

Study procedure and questionnaire design

This survey was distributed to participants via online social media networks, such as Facebook, Twitter, Instagram, Snapchat, and WhatsApp. This survey was adapted from previously published studies in light of the current literature [9].

The self-administered questionnaire was translated into the Arabic language by two independent bilingual translators, and then a panel of experts and translators reviewed the translation to resolve discrepancies. A reverse translation from Arabic to English was also performed to guarantee that there were no errors in the investigation tool. Ten respondents reviewed the questionnaire before the survey and provided feedback that led to the simplification of specific medical terminology and the revision of several questionnaire items.

The questionnaire comprised 25 close-ended questions in two sections. The first section included seven questions on the participants’ sociodemographic information, while their understanding of cancer genetics and genetic testing was assessed in the second section, which included 18 questions of the response type yes/no/don’t know.

To maintain anonymity and confidentiality, the participants’ names, phone numbers, and identity card numbers were not included. Each respondent provided informed consent online before taking the survey after we assured them that the survey was voluntary, confidential, and intended only for academic purposes.

Statistical analysis

The final data were entered into Microsoft Excel spreadsheets to check for completeness and minor typographical errors. Next, descriptive statistics were expressed as percentages for categorical variables, and the mean and standard deviation (SD) for continuous variables were taken using SPSS 23 software (IBM, Armonk, NY). Finally, the categorical variables were computed using the independent chi-square test to determine all relevant knowledge-related factors. A p-value of ≤ 5% was considered significant.

A modified Bloom’s criteria scoring system was used to analyze the data coded and inputted into SPSS 23 software. For questions regarding knowledge of genetic testing, a score of 1 was given for “yes” and a score of 0 for “no” and “don’t know.” The total score was then summed and classified into the following categories: 80%-100% was considered as good, 50%-79% as moderate, and less than 50% as poor [12].

## Results

In this cross-sectional survey, we enrolled 1329 participants in Mecca Province, with a 93.20% response rate. The participants’ mean age was 33.4 (SD=13.4, Table 1). The largest group of participants was 21-30 years old (n=524, 39.4%), while those over 60 years old constituted the smallest group (n=41, 3.1%). Most of the responses were from females (n=799, 60.1%). There were more responses from Mecca City (n=599, 45.1%) than from any other city in Mecca Province (Table 1). Most participants had a university degree (n=838, 63.1%), and single and married participants were close to equally represented (n=643, 48.4% and n=613, 46.1%, respectively) (Table 1). Concerning the participants’ history of cancer, about 5.4% had previously been diagnosed with cancer, and about 20.4% had a family history of cancer (Table 1).

**Table 1:** Participants’ demographics (n=1,329)

Categories		N	%
Age groups	18-20	166	12.5
	21-30	524	39.4
	31-40	237	17.8
	41-50	239	18.0
	51-60	122	9.2
	˃ 60	41	3.1
Gender	Male	530	39.9
	Female	799	60.1
Residence	Taif	310	23.3
	Al Qunfudhah	103	7.8
	Al Laith	10	0.8
	Jeddah	294	22.1
	Rabigh	7	0.5
	Makkah	599	45.1
	Maysaan	6	0.5
Educational level	High school	264	19.9
	University	838	63.1
	Postgraduate education	153	11.5
	Other	74	5.6
Marital status	Single	643	48.4
	Married	613	46.1
	Other	73	5.5
Past history of cancer	Yes	72	5.4
	No	1257	94.6
Family history of previous cancer	Yes	271	20.4
	No	1058	79.6
Age, Mean ± Standard deviation (SD)	Mean=33.4, SD=13.4		

**Figure 1:**
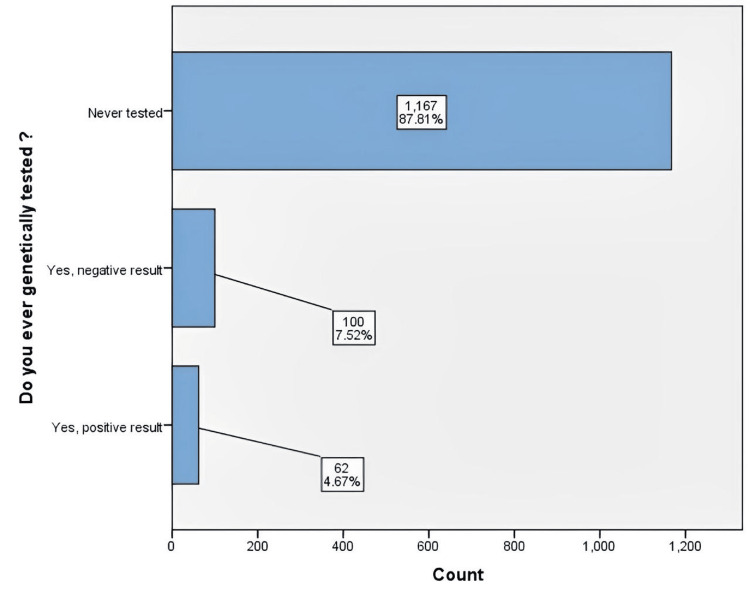
Cancer genetics testing status among the participants (n=1,329)

Most of the participants (n=1167, 87.81%) had never undergone cancer genetics testing, but about 4.67% had been tested with a positive result and 7.52% with a negative result (Figure 1).

Table 2 shows the participants’ answers regarding cancer genetics general knowledge. Most of the participants had an insufficient level of knowledge regarding the concept of inherited risk for cancer, as shown in items 1, 2, 5, 14, and 16 (17.8%, 36.5%, 43.4%, 32.3%, and 29.9%, respectively). Moreover, the participants answered items 4, 7, 10, 11, 12, 13, 15, 17, and 18 incorrectly, representing 58.5%, 50.6%, 72.0%, 50.2%, 59.2%, 64.0%, 57.2%, 59.9%, and 64.2%, respectively. However, the participants’ correct responses to items 3, 6, 8, and 9 represent 62.8%, 50.6%, 67.0%, and 56.9%, respectively (Table 2).

**Table 2:** Frequency of participants’ responses (n=1,329)

Items	Categories		N	%
1	Most people who develop cancer do so because they have an inherited risk for cancer. (Incorrect)	Yes	564	42.4
		No	236	17.8
		Don’t know	529	39.8
2	A person with an inherited risk for cancer will definitely get cancer one day. (Incorrect)	Yes	350	26.3
		No	485	36.5
		Don’t know	494	37.2
3	People with an inherited risk for cancer (and their at-risk relatives) are more likely to develop more than one type of cancer. (Correct)	Yes	834	62.8
		No	106	8.0
		Don’t know	389	29.3
4	Knowing about an inherited risk (passed down within a family) can affect choices about cancer treatments (for example, medications or surgery). (Correct)	Yes	551	41.5
		No	196	14.7
		Don’t know	582	43.8
5	If an inherited risk for cancer is found, there is nothing a person can do to change his/her cancer risk (Incorrect)	Yes	234	17.6
		No	577	43.4
		Don’t know	518	39.0
6	The lifetime chance of getting cancer depends on which altered cancer gene is inherited. (Correct)	Yes	673	50.6
		No	119	9
		Don’t know	537	40.4
7	People with an inherited risk for cancer may get cancer at a younger age than people with an average risk. (Correct)	Yes	656	49.4
		No	128	9.6
		Don’t know	545	41
8	A person with an inherited risk for cancer may be advised to start cancer screening at an earlier age than people with an average chance. (Correct)	Yes	891	67
		No	82	6.2
		Don’t know	356	26.8
9	People who have inherited cancer risk may be recommended to get special cancer screening or risk-reducing surgery. (Correct)	Yes	756	56.9
		No	109	8.2
		Don’t know	464	34.9
10	Female-specific cancer risk, such as ovarian cancer, can generally be passed on from either the father or mother. (Correct)	Yes	373	28.1
		No	231	17.4
		Don’t know	725	54.6
11	The blood relatives (for example, sister, father, or child) of a person with a mutation in a cancer-risk gene might share the same gene mutation. (Correct)	Yes	662	49.8
		No	147	11.1
		Don’t know	520	39.1
12	A person with an inherited risk for cancer may have distant relatives (for example, cousins) who also have increased cancer risk. (Correct)	Yes	542	40.8
		No	176	13.2
		Don’t know	611	46
13	If a genetic test does not identify an inherited risk for cancer now, there is a chance that a mutation could be identified through future tests. (Correct)	Yes	478	36
		No	166	12.5
		Don’t know	685	51.5
14	All children of a person with inherited cancer risk will also have an inherited cancer risk. (Incorrect)	Yes	322	24.2
		No	429	32.3
		Don’t know	578	43.5
15	In most cases, the sisters and brothers of a person with inherited cancer risk have a 50-50 (50%) chance of having an inherited risk for cancer too. (Correct)	Yes	568	42.7
		No	173	13
		Don’t know	588	44.2
16	All of the gene mutations that could increase the risk for cancer have been discovered. (Incorrect)	Yes	258	19.4
		No	397	29.9
		Don’t know	674	50.7
17	Some gene mutations mean a larger increase in the risk for cancer while others mean a smaller increase in the risk for cancer. (Correct)	Yes	532	40
		No	128	9.6
		Don’t know	669	50.3
18	The effects of all gene mutations on cancer risks are not known at the current time. (Correct)	Yes	476	35.8
		No	135	10.2
		Don’t know	718	54

**Figure 2:**
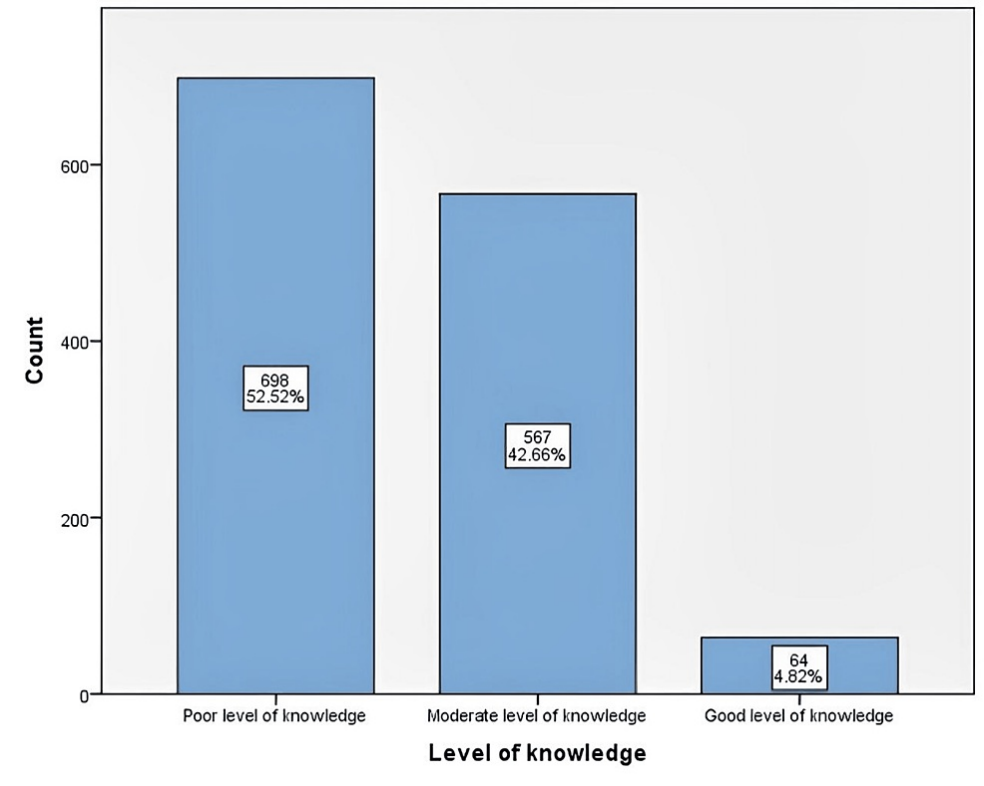
Overall level of knowledge about cancer genetics and the importance of genetic testing (n=1329)

The participants with an overall inadequate knowledge score constituted 52.52% of the sample, whereas 42.66% demonstrated a moderate level of understanding. Only 4.82% of the participants showed a good level of understanding (Figure 2).

Table 3 shows the associations between the participants’ demographic data and their knowledge scores. Participants aged 21-30 years and those with university degrees corresponded significantly with all levels of knowledge scores (p-value˂.001). Single participants showed significant variation between a good level (n=49) and a moderate level of knowledge (n=305), while married participants corresponded significantly with a poor level of understanding (n=358; p-value˂.001). Additionally, participants who had never been genetically tested for cancer corresponded significantly with all levels of knowledge scores (n=60, n=478, and n=629, respectively; p-value=.011). By contrast, the participants’ gender, residence, and past medical and family history showed no significant association with the level of knowledge scores (p-values=.263, .107, .116, and .733, respectively; Table 3).

**Table 3:** The association between the participants’ demography and level of knowledge scores

Categories		Level of knowledge			P-value
		Good level of knowledge (N)	Moderate level of knowledge (N)	Poor level of knowledge (N)	
Age groups	18-20	9	71	86	˂0.001*
	21-30	43	257	224	
	31-40	3	92	142	
	41-50	5	89	145	
	51-60	3	46	73	
	˃ 60	1	12	28	
Gender	Male	28	238	264	0.263
	Female	36	329	434	
Residence	Taif	8	128	174	0.107
	Al Qunfudhah	7	57	39	
	Al Laith	1	4	5	
	Jeddah	16	113	165	
	Rabigh	0	2	5	
	Makkah	32	261	306	
	Maysaan	0	2	4	
Educational level	High school	8	110	146	˂0.001*
	University	51	386	401	
	Above university	5	48	100	
	Other	0	23	51	
Marital status	Single	49	305	289	˂0.001*
	Married	14	241	358	
	Other	1	21	51	
Past history of previous cancer	Yes	0	35	37	0.116
	No	64	532	661	
Family history of previous cancer	Yes	14	110	147	0.733
	No	50	457	551	
Do you ever genetically tested?	Yes, positive result	2	31	29	0.011*
	Yes, negative result	2	58	40	
	Never tested	60	478	629	

## Discussion

Cancer represents a worldwide burden as one of the leading causes of death, killing approximately 10 million people in 2020 [1]. Moreover, a recent study in the KSA reports that the incidence of some types of cancer has increased threefold in recent years because of numerous modifiable factors, such as a poor level of cancer awareness, a lack of screening and early detection programs, social barriers against cancer diagnostic investigations, and a lack of screening for genetic predisposition [2]. Therefore, this cross-sectional study investigated the general population’s knowledge of cancer genetics to highlight the importance of genetic testing.

Our findings show that 52.52% of the total participants had a poor level of knowledge about cancer genetics and the importance of genetic testing, whereas the rest of the respondents demonstrated moderate to good levels of knowledge. Furthermore, 20.4% of the participants had a family history of cancer, and 5.4% had a personal history of cancer. The majority of those with a personal or family history of cancer showed a low level of awareness. Consistent with our findings, a study among females diagnosed with breast cancer and their relatives in a Nigerian teaching hospital found an insufficient awareness of cancer genetics, and a majority of the participants had poor knowledge about the genetics of breast cancer [13].

Another study, conducted among Saudi women, found a suboptimal awareness of inherited and familial cancers, with an apparent influence of increased age on their knowledge level [14]. By contrast, the majority of our respondents who demonstrated lower levels of knowledge were 21-30 years old and were female. As was reported in a recent study among females in Mecca, KSA, this lack of knowledge can be a major contributing factor to the lack of proper screening measures for some types of cancer, such as breast cancer, among individuals at risk [15].

Multiple studies have confirmed that consanguineous marriage is associated with an increased risk of certain types of cancer, such as colorectal, prostate, lymphoma, and leukemia [16]. The KSA has a high rate of consanguinity at nearly 56% [17], but our findings indicate that the married respondents had a predominantly poor level of knowledge of the concept of inherited cancer risk, which obviously demonstrates the need for awareness campaigns and programs to enhance the understanding of the general population and especially of those who are about to be married about genetic predisposition to cancer.

The identification of mutated genes through genetic tests is crucial, as it can provide patients and their relatives with the estimated risk of cancer among the mutation carriers to establish proper surveillance and/or preventive procedures [6]. Nevertheless, most of our survey respondents had never been genetically tested, although 7.52% had been tested with negative results and 4.67% had been tested and identified as having a genetic predisposition to cancer.

A recent study has identified factors that may affect the level of cancer genetics awareness, including higher education and medical literacy [18]. This supports the need for additional educational programs to clarify the scientific importance and medical benefits of genetic testing to the general population.

The limitations of this study could be mitigated by minimizing the variations between the sociodemographic categories of the participants, such as age group and gender, to enable researchers to accurately compare the findings and identify the related factors influencing the results. Additionally, the assessment of the participants’ past history of cancer and the results of their genetic tests should be according to medical data rather than self-reported data.

## Conclusions

The early identification of mutated genes through genetic tests is essential since it can inform patients and their relatives about their genetic predisposition to cancer, which in turn enables proper surveillance and/or preventive procedures. Thus, this study investigated the knowledge of cancer genetics and of the importance of genetic testing among the general population of Mecca Province, KSA.

Our findings indicate that 52.52% of the participants had a low level of knowledge, while the remainder demonstrated a moderate to good level of knowledge. Accordingly, there is clearly a need for awareness campaigns and programs to improve the general population’s understanding as well as additional educational programs to explain the scientific importance and medical benefits of genetic testing to the general population.
